# The next generation

**DOI:** 10.1093/jxb/erae076

**Published:** 2024-03-27

**Authors:** John E Lunn

**Affiliations:** Editor in Chief, JXB Max Planck Institute of Molecular Plant Physiology, 14476, Potsdam-Golm, Germany

**Fig. 1.**
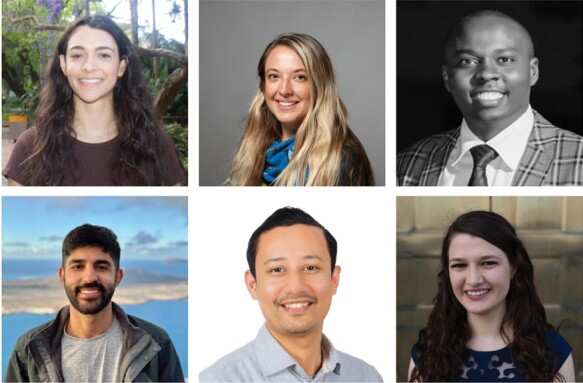
Introducing *JXB*’s Editorial Interns. Top (L to R): Hannah Drieberg, Tessa Reid, Martin Mburu. Bottom (L to R): Diego Pinheiro Brito, Sujit Jung Karki, Natalie Hoffmann.

**Following the success of our inaugural editorial internship programme in 2023, the *Journal of Experimental Botany* is pleased to announce the appointment of six early career researchers as editorial interns for 2024: Diego Pinheiro Brito (Max Planck Institute of Molecular Plant Physiology, Germany), Hannah Drieberg (University of Queensland, Australia), Natalie Hoffmann (University of Toronto, Canada), Sujit Jung Karki (University College Dublin, Ireland), Martin Mburu (University of Hannover, Germany), and Tessa Reid (Rothamsted Research, UK) (** **Fig. 1** **). The aim of these internships is to help train the next generation of editors.**

Our interns have each been paired with an editor who shares their scientific interests and will serve as a mentor during their one-year internship. The editor and intern will work together on selected manuscripts through all stages of the editorial review process, from initial assessment and selection of reviewers to the final evaluation and decision. Interns will abide by the strict rules on confidentiality that apply to editors and reviewers, and the final decision on each manuscript will remain the sole responsibility of the Handling Editor. In addition to gaining experience of editorial work, our interns will also be invited to write Insight commentaries on selected papers and provide content for *JXB’s* social media channels. If you would like to know more about our editorial interns, please visit the *JXB*website. I would like to thank our 2023 editorial interns – Francesca Bellinazzo, Konan Ishida, Nishat Shayala Islam, Chao Su, Catherine Walsh, and Arpita Yadav – for their valuable contributions to the work of the journal over the last year, and we wish them every success in their future careers.

*JXB* was founded by the Society for Experimental Biology (SEB), and continues to be owned and managed by the SEB for the benefit of the science community. A key mission of the SEB is to help young scientists to make their way in the science world, by providing opportunities to meet and exchange ideas with other scientists at SEB conferences. The SEB also organises training and careers workshops for early career researchers, and our editorial internship programme was inspired by this ethos.

After celebrating the SEB’s Centenary in 2023, we are now looking forward to another landmark in 2025, which will see the 75th anniversary of the foundation of *JXB* in 1950. In partnership with the SEB, we are planning a 75th Anniversary meeting that will provide an opportunity for early career researchers to meet and share their work and vision for the future of plant science. Further information about the *JXB* 75th Anniversary meeting will be posted on the *JXB* and SEB websites as details are confirmed, so watch this space.

